# New Challenges of Treatment for Locally Advanced Head and Neck Cancers in the Covid-19 Pandemic Era

**DOI:** 10.3390/jcm10040587

**Published:** 2021-02-04

**Authors:** Camil Ciprian Mireștean, Anda Crișan, Adina Mitrea, Călin Buzea, Roxana Irina Iancu, Dragoș Petru Teodor Iancu

**Affiliations:** 1 Department of Oncology and Radiotherapy, University of Medicine and Pharmacy Craiova, 200349 Craiova, Romania; mc3313@yahoo.com (C.C.M.); anda_crisan2005@yahoo.com (A.C.); 2 C.F. Clinical Hospital, 700506 Iași, Romania; 3 County Clinical Emergency Hospital Craiova, 200642 Craiova, Romania; ada_mitrea@yahoo.com; 4 Diabetes, Nutrition and Metabolic Diseases, University of Medicine and Pharmacy Craiova, 200349 Craiova, Romania; 5 National Institute of Research and Development for Technical Physics, 700050 Iaşi, Romania; calinb2003@yahoo.com; 6 “Prof. Dr. Nicolae Oblu” Emergency Clinic Hospital, 700309 Iaşi, Romania; 7 Department of Oral Pathology, Gr. T. Popa, University of Medicine and Pharmacy, 700115 Iaşi, Romania; 8 Department of Clinical Laboratory, St. Spiridon, Emergency Hospital, 700111 Iaşi, Romania; 9 Department of Oncology and Radiotherapy, “Gr. T. Popa” University of Medicine and Pharmacy, 700115 Iaşi, Romania; dt_iancu@yahoo.com; 10 Regional Institute of Oncology, 700483 Iaşi, Romania

**Keywords:** head and neck cancer, non-surgical, radiotherapy, chemotherapy, Covid-19

## Abstract

Locally advanced head and neck cancer is a unique challenge for cancer management in the Covid-19 situation. The negative consequences of delaying radio-chemotherapy treatment make it necessary to prioritize these patients, the continuation of radiotherapy being indicated even if SARS-CoV-2 infection is confirmed in the case of patients with moderate and mild symptoms. For an early scenario, the standard chemo-radiotherapy using simultaneous integrated boost (SIB) technique is the preferred option, because it reduces the overall treatment time. For a late scenario with limited resources, hypo-fractionated treatment, with possible omission of chemotherapy for elderly patients and for those who have comorbidities, is recommended. Concurrent chemotherapy is controversial for dose values >2.4 Gy per fraction. The implementation of hypo-fractionated regimens should be based on a careful assessment of dose-volume constraints for organs at risks (OARs), using recommendations from clinical trials or dose conversion based on the linear-quadratic (LQ) model. Induction chemotherapy is not considered the optimal solution in this situation because of the risk of immunosuppression even though in selected groups of patients TPF regimen may bring benefits. Although the MACH-NC meta-analysis of chemotherapy in head and neck cancers did not demonstrate the superiority of induction chemotherapy over concurrent chemoradiotherapy, an induction regimen could be considered for cases with an increased risk of metastasis even in the case of a possible Covid-19 pandemic scenario.

## 1. Introduction

The disease caused by the new coronavirus identified in Wuhan, China, Hubei province, has spread rapidly to all continents, causing a pandemic with severe consequences. The introduction of preventive measures including emergency restrictions on citizens’ freedoms, implementation of social distancing and wearing personal protective equipment (PPE) has limited the number of infections in most countries, a lesson learned from the experience of pandemic epicenters in China and Lombardy in Italy. After the pandemic epicenter in summer 2020 was Latin America, now India is tending to become the hottest spot in a second wave of infections affecting the whole world. Leung et al. draw attention to the increase in the rate of transmission of the SARS-CoV-2 virus associated with a possible second wave of infection in case of premature relaxation of the restrictions. On 1 December, 6,392,290 cases had been diagnosed since the beginning of the pandemic, of which 1,480,662 patients died and 44,230,469 were cured. Cancer patients were considered a high-risk group in developing severe forms of the disease with potential for worse outcome [1,2,3].

Among cancer patients, those diagnosed with head and neck cancers offer the multidisciplinary team one of the most extreme diagnostic and treatment challenges during the Covid-19 pandemic. The definition of locally advanced head and neck cancers generally refers to advanced tumor (T) and nodal (N) stages. Locally advanced head and neck cancer is a unique challenge for cancer management in the Covid-19 scenario, taking into account the complexity of the treatment that requires the involvement in at least one of the stages of treatment of both the head and neck surgical oncologist, the medical oncologist and the radiation oncologist. Although non-surgical treatment is the most common choice for these stages of disease evolution, the development of resection and reconstruction techniques has opened new horizons. Surgical treatments and the term “unresectable” are not currently superimposed on the “locally advanced” disease concept. Due to the risk of aerosolization associated with upper aerodigestive tract investigations taking into account the presence of virus in the respiratory mucosa of the naso- and oropharynx, but also due to increased mortality, the management of head and neck cancers is a challenge during a Covid-19 outbreak. Aerosolized droplets expelled during breathing, coughing or sneezing have a high risk of transmitting the disease. 85% of health care workers are exposed to SARS-CoV-2 infection, even if they wear a N95 filter mask which can provide adequate protection in this situation. Radiotherapy is essential as a treatment method, part of a multidisciplinary approach to locally advanced head and neck cancers offering a curative potential therapeutic approach. The physical presence of head and neck cancer patients in the radiotherapy department for approximately seven weeks significantly increases the risk of exposing them to a SARS-CoV-2 infection [3,4,5].

## 2. Locally Advanced Head and Neck Cancer Treatment in Covid-19 Pandemic Outbreak—A Dance on a Slack Wire

Comorbidities coexist with head and neck cancers in 36.4–88.9% and 65–90% of cases diagnosed at an advanced stage. Being mostly heavy smokers, chronic obstructive pulmonary disease (COPD) is one of the most common comorbidities in this group of patients. All these factors significantly increase the risk of a severe outcome, including acute respiratory distress syndrome (ARDS), hospitalization in intensive care units (ICUs), mechanical ventilation, and even death. Sidaway et al. report 53.6% of cancer patients requiring mechanical ventilation and a 28.6% death rate in this category of patients, with most fatalities due to ARDS. Another specific aspect of head and neck cancer (HNC) is immunosuppressive treatment, an effect that is associated with a compromised immunity state generated by cancer, thus increasing the risk of a worse outcome in case of infection with the new coronavirus [6,7,8].

In a systematic review and meta-analysis that evaluated the effect of oncological therapies on the course of Covid-19 disease, Yekedüz et al. identified chemotherapy as a risk factor for SARS-CoV-2 infection mortality without a consensus on safety in surgery, radiation therapy, target molecular therapy, and immunotherapy. A systematic review and meta-analysis by Wang et al. concluded that these therapies do not increase the risk of death in combination with Covid-19, chemotherapy being in this case too considered the only cancer therapy at risk. Cancer is considered due to its immunosuppressive status a risk factor in association with SARS-CoV-2 infection, the rate of possible severe complications, not only ARDS, considered to be 33%. In the new context created by the Covid-19 pandemic, it is necessary to adapt the treatment guidelines in order to limit as much as possible the risk of infection, both for the medical staff and the patients, given the unpredictable variation of hospital resources, as well as the fluctuation of the spread of the virus, expected to have an “in waves” evolution for several months or even longer. The effect of the pandemic is disproportionate, the most affected being the countries with health systems that will become overburdened, with the risk of exacerbating socioeconomic inequalities. There is a consensus on the protection of patients and medical staff, the use of telemedicine services is recommended in the departments involved in the treatment of head and neck cancers, and an individualized decision should be taken after assessment of the benefit and risk of treatment, whether it is surgical followed by adjuvant treatment, or definitive radio-chemotherapy [9,10,11].

An international multicenter study that included 1137 patients treated with curative intent during the Covid-19 pandemic identified, in the group analyzed, positive SARS-CoV-2 test rates of 3% and an overall mortality of 1.2% 30 days after surgery. Tracheostomization and oral tumor surgery is considered to be at increased risk of staff contamination, a factor that cannot be overlooked given the reportedly unfavorable pathways, the high death rate among otolaryngologists and head neck surgeons being probably correlated with a high viral load. The authors consider the non-surgical treatment of cancers of the salivary glands and oral cavity suboptimal, considering only for oropharyngeal and laryngeal cancers the option of treatment with radio-chemotherapy. However, the lung complication rate of 51.2% with a death rate of 23.8% is another argument that advocates avoiding surgery in the upper aerodigestive tract [12,13,14].

SARS-CoV-2 has had effects on HNC management in choosing optimal treatment, taking into account that resources available in each department involved are essential. The multidisciplinary team (MDT) has an essential role in evaluating the optimal option and is also involved in patient evaluation and follow-up during this pandemic period [15,16].

Bowman et al. consider the Covid-19 pandemic to be similar to Hurricane Katrina, with reduced access to cancer care following this disaster. During these periods, there is an argument that the evaluation of a patient should be delayed. Limiting hospital visits by using telemedicine services, using PPE including face masks and patient testing, and reducing patients’ stay time in the waiting room through telephone scheduling are strategies that can limit the risk of contamination during radiotherapy treatment. Patients with dysphagia and dyspnea require percutaneous gastrostomy or tracheostomy tubes which have an increased risk of aerosolization and spread of the virus, so testing HNC patients and staff involved in their treatment is a priority. For these reasons, given the difficulties in identifying cough as a consequence of oncological disease or as determined by Covid-19 infection, H&N and lung cancers patients require triage and correct evaluation of the etiology of symptoms. The need to travel daily for long distances and possible lack of telemedicine services create additional difficulties in providing health care to HNC patients. This situation is exacerbated by the fear of patients not contacting treatment services, which has the consequence of delaying treatment. Considering mortality is in the 40–50% range, much higher than Covid-19 mortality estimated at 1–2% in the United States, HNC treatment is a priority. However, the high mortality rate in cancer patients who become infected with the new coronavirus must be taken into account. Afshar et al. consider the fatality rate as between 5.5% and 60% in this group of patients without specifying the type of cancer [17,18,19,20,21,22].

Surgery services are the most affected by the risk of contaminating staff when treating patients with HNC, given the need for procedures that require aerosolization. Under these conditions, it is recommended to delay the treatment of benign tumors, but also of low-grade carcinomas such as mucoepidermoid carcinoma, acinar cell carcinoma and polymorphic adenocarcinoma as long as the COVID-19 pandemic exposes patients and medical staff to the risk of contamination. In the case of low grade malignancies, follow-up by computer tomography (CT) or magnetic resonance imaging (MRI) is recommended [19].

## 3. Covid-19 “Tsunami”—The Urgent Need for Recommendations

The standard treatment with potentially curative effect for locally advanced HNC is concomitant chemo-radiotherapy. Any delay of treatment increases the risk of death by 16% for each month. A protocol for radiotherapy in the case of a Covid-19 pandemic outbreak has been proposed by a panel of international experts from the American Society of Radiation Oncology (ASTRO) and the European Society of Radiation Oncology (ESTRO). Cutoffs chosen for strong agreement and agreement were 80% and 66%, respectively, taking into account two possible pandemic scenarios.

The first, “early” scenario of risk mitigation is characterized by the aim to reduce risk infection for patients and staff and also focuses on the evaluation of serious infection risk for patients receiving chemotherapy and radiation therapy. This scenario takes into account the necessity to avoid repeated hospital visits. In this scenario, the risk of complications and severe toxicities associated with intensive chemo-radiotherapy treatments is also a cause of concern.

In the second “late” scenario, the resources of radiotherapy services are severely reduced and some patients cannot benefit from treatment.

A panel of experts chose five different cases for which they proposed a multidisciplinary approach. For all cases that were proposed for definitive non-surgical treatment, the experts voted by “strong agreement” not to postpone treatment except for a T1b node negative glottic cancer case. In this case the consensus vote was validated by “agreement”. Oropharyngeal Human Papilloma Virus (HPV) positive (+) and negative (−) and locally advanced larynx cancer were considered as having a high priority for treatment, followed by the T1N0 glottic larynx cancer. In a very limited resource scenario, HPV+ oropharynx was considered a top priority and locally advanced larynx was chosen before HPV-oropharynx cases for priority treatment. By positive margins, operated oral cavity cases were prioritized before early glottic cancer. SARS-CoV-2 infection and symptomatic benefit, followed by curative option, were the criteria considered relevant for the beginning of treatment in less than one week. Not postponing HNC radiotherapy for more than four–six weeks and considering radical treatment for these cases as a high priority were recommendations with “strong agreement”. For all cases proposed for radio-chemotherapy treatment, there is a consensus to postpone treatment in the case of SARS-CoV-2 infections until a negative real-time polymerase chain reaction (RT-PCR) test. The consensus was not to discontinue treatment if the patient had started radiotherapy treatment at the time of Covid-19 diagnosis. If, during treatment, the patient presents cough, chest pain, or requires oxygen support due to respiratory problems, the consensus is to discontinue treatment, considering the risk of hospitalization in the ICU department with a potentially fatal evolution, the risk in this case being considered higher than the benefit of radio-chemotherapy. A 10–14 day waiting period is recommended after repeated negative tests for this category of patient before treatment restarts. The risk of contaminating staff or other patients, the possibility of worsening of the general condition with the need for an emergency room presentation and the need for a feeding tube may be arguments for discontinuation the treatment in patients diagnosed with SARS-CoV-2 with mild symptoms. A cutoff of two weeks from the start of treatment is considered significant in order not to interrupt treatment in case of SARS-CoV-2 infection, in the case of mild symptoms without any additional risks.

Regarding the fractionation scheme in the first pandemic scenario, there was a strong agreement to keep the same fractionation scheme in all cases proposed for potentially curative treatment. In the scenario of limited resources, hypofractionation was proposed with a strong agreement consensus. Cisplatin-based chemotherapy 80–100 mg/m^2^ every three weeks or 30–40 mg/m^2^ weekly are the most common regimens for a concurrent approach, with a preference 6/4 ratio for the three-week regimen. If in the first scenario there was consensus for chemotherapy administration in all cases that had guideline indications, in the scenario of limited resources there was a consensus to de-escalate chemotherapy for HPV+ oropharyngeal cancer, but for other cases there were opinions on omitting concomitant chemotherapy. Conventional radiotherapy or moderate hypofractionation with a maximum 2.4 Gy per fraction were accepted in concurrent radio-chemotherapy protocol.

Induction chemotherapy was not considered as an option by most experts, only 10% considering this as a therapeutic standard, and only 27% considering it as an option for the timing of radiotherapy. The rationale for avoiding induction chemotherapy was based on the immunosuppressive potential associated with an increased risk of contracting Covid-19 disease, but also of developing severe clinical evolution to ARDS or even death [19,20].

## 4. Discussion

COVID-19, caused by a new coronavirus, has created a global pandemic, with an average mortality rate associated with this disease of about 3.0%. In mode of transmission and clinical manifestations, SARS-CoV-2 resembles other coronaviruses (MERS-CoV and SARS-CoV) that have caused severe outbreaks. One of its peculiarities is the destruction of the lung parenchyma following a cytokine storm, a phenomenon observed especially in the elderly and associated with immunosuppressive factors. Without an effective specific treatment to date, vaccines based on the revolutionary mRNA technology which encodes the spike protein (S protein) of SARS-CoV-2 open new horizons in infection prevention and blocking new pandemic waves. With a median reported rate of severe complications of 33% including respiratory failure, lung injury, acute kidney failure and severe pneumonia, cancer patients are considered a vulnerable category to SARS-CoV-2 infection. Not only is the rate of severe complications a risk, but cancer patients have a two-fold higher risk than the general population of contracting SARS-CoV-2 infection. Vaccination of cancer patients is an absolute priority in this context, although the interactions between vaccines and cancer and the protective effect on immunocompromised patients is still poorly understood. The priority vaccination strategy for cancer patients is all the more justified as new mutations in the coronavirus, including the British mutation, tend to be much more contagious, but there is no evidence that these mutations produce more severe forms of the disease. Although the vaccines approved so far appear to be active on these SARS-CoV-2 mutations, the scientific community is on alert for the risk of a spike protein mutation that would make the vaccinated population vulnerable to a new pandemic wave. The need to stop the spread of possible new mutations in the virus makes it necessary to partially maintain the lockdown measures and especially to maintain the rules of individual protection and social distance, even in the context of a mass vaccination of the population [23,24,25,26,27,28].

Although in particular cases anti-cancer treatments may have protective effects on SARS-CoV-2 infection, it is generally accepted that cancer is associated with pro-inflammatory status and immunosuppression. For example, androgen depletion therapy (ADT) may reduce the risk of infection by down-regulation of the serine protease for the S protein, responsible for the entry of SARS-CoV-2 into the cell. Irradiation of the pulmonary parenchyma with low doses may reduce inflammation mediated by the hyperactivated immune response, the use of ionizing radiation in the treatment of pneumonia having historical precedents. However, oncological surgery and chemotherapy are associated with lymphopenia and immunosuppression, being an additional factor in the increased risk of SARS-CoV-2 infection and the development of severe forms of disease among cancer patients [29,30,31,32,33].

Regardless of which scenario will be considered as therapeutic option, the analysis of the case in the MDT is essential, and an assessment of the risk of any possible needed surgical procedure or admission to the ICU must be performed. Any therapeutic decision must take into account the possibility of no hospital bed availability or limited technical possibilities to safely perform interventions, such as percutaneous gastrostomy or tracheostomy. As demonstrated by Hintze et al., the postoperative mortality of HNC cases diagnosed with SARS-CoV-2 is increased, the authors reporting two deaths in three cases diagnosed with Covid-19 in patients who required surgery. The authors demonstrate that patients with HNC are not vulnerable, but postoperative risk for SARS-CoV-2 infection is increased by the prolonged hospitalization time and the need for interaction with a relatively large number of healthcare workers. In this context, a balanced therapeutic attitude focused on a curative option, limiting the time spent by the patient in the department but also avoiding complications that may require surgical or aerosolization procedures is the most rational approach [34].

The ability to implement hypo-fractionated radiotherapy programs will take into account the experience of the department in using altered fractionation schemes and the correct evaluation by the radiation oncologist, medical physicist and radiobiologist of modifying dosimetric constraints for such a treatment scheme. Portaluri et al. consider the rapid implementation of hypo-fractionated radiotherapy “a mix of evidence based medicine and opportunities”, mentioning the risk involved in adopting this fractionation protocol if there is no solid scientific evidence, but also the advantages that hypo-fractionated radiotherapy brings in the context of the need to expose patients during treatment sessions, but also to limited treatment capabilities. In the case of HNC, the hypo-fractionated irradiation regimen was not included in the meta-analyzes and the phase 3 trials analyzing altered fractionation radiotherapy did not include a study arm with hypofractionation [35,36].

There are two different alternatives in adapting the dose volume recommendation: choosing the dosimetric constraints proposed and validated in clinical trials; or using the linear-quadratic model (LQ) for normal tissue included as organs at risks (OARs), an α/β ratio = 3 or even a value of 2 for the calculation of the toxic dose for spinal cord and the risk of radio-induced myelopathy. In the context created by the new coronavirus pandemic, hypo-fractionated radiotherapy has become one of the main indications for cancer management, using radiotherapy in a limited resource scenario. In the case of cancers characterized by low values of α/β ratio such as breast and prostate cancer, hypofractionation is recommended as standard treatment, as rapidly proliferating tumors such as HNC and cervical cancer benefit from the advantages of conventional fractionation. The possibility of new radiotherapy techniques with modulated intensity to deliver different doses of radiation per fraction in different sub-volumes, i.e., the simultaneous integrated boost (SIB) technique, creates the premises for the significant reduction of the number of fractions in HNC radiotherapy, having a possible radiobiological benefit of slightly increased dose per fraction for radioresistant sub volumes or tumors [37,38,39].

The optimal Intensity Modulated Radiotherapy (IMRT) technique is currently a therapeutic standard in HNC radiotherapy, having the ability to provide better conformation of target volumes and superior protection of OARs, especially parotid glands, with clinical consequences in reducing xerostomia and improving the quality of life of HNC patients. If the IMRT technique using standard fractionation demonstrated clinical results in terms of tumor control equivalent to the 3D conformal radiotherapy (3D-CRT) technique, or even superiority in term of local control and OS for nasopharyngeal cancers radio-chemotherapy, the more recently proposed technique using SIB was clinically validated by the meta-analysis of Jiang et al. [40].

For HNC, the optimal SIB regimen has not yet been determined. The authors compared the clinical results of simultaneous integrated pulse (SIB)-IMRT versus sequential pulse (SEQ)-IMRT in HNC. A comparative analysis between SIB-IMRT and sequential boost IMRT showed no significant difference in measuring overall survival (OS), progression free survival, free survival with locoregional recurrence and distant metastases free survival. No increased toxicity rates associated with the use of SIB were reported. The volumetric intensity modulated arc therapy (VMAT) technique was considered clinically and dosimetrically equivalent to IMRT, presenting a possible radiobiological advantage by reducing the overall delivery time of the treatment. SIB-IMRT or SIB-VMAT can reduce the definitive chemo-radiotherapy treatment for locally advanced HNC to 30–33 fractions [31,32,33,34,35,36,37,38,39,40,41,42,43,44,45].

Concurrent radio-chemotherapy treatment is considered the therapeutic standard in locally advanced NHC. However, the addition of platinum-based chemotherapy increases the rate of acute and late toxicities, and discontinuations of treatment for more than a week can lead to the loss of approximately 4–8% benefit in OS. A meta-analysis by Pignon et al. reports a median benefit of 6.5% for concurrent radio-chemotherapy. Cisplatin alone or cisplatin/carboplatin associated with 5-fluorouracil (5-FU) or other combination of poly-chemotherapy that includes a platinum agent have been shown to be equally effective in concurrent use with radiation therapy [46].

The use of any chemotherapy agent other than Cisplatin alone is inferior to Cisplatin monotherapy. The use of any other protocol that includes Carboplatin is considered a strong immunosuppressant and in this epidemiological context the substitution of Cisplatin with Carboplatin is not recommended. Although considered difficult to tolerate and with a higher toxicity rate than the 40 mg/m^2^ weekly protocol, the three-week Cisplatin regimen brings the advantage of reducing the number of visits to the medical oncology department with a consequence of additional exposure to a risk of SARS-CoV-2 infection. Meta-analysis of chemotherapy in head and neck cancers (MACH-NC) demonstrates a reduction in the benefit of concomitant chemotherapy for elderly patients due to the possible effect of dilution, the death rate among these patients from other medical causes than cancer being higher.

Regarding the use of induction chemo-therapy the results are controversial. Haddad et al. demonstrate the superiority of the taxane, platinum, fluorouracil (TPF) regimen versus platinum doublet as induction chemotherapy in HNC. Induction chemotherapy may reduce the rates of metastasis-free survival in HPV-oropharyngeal cancers, as demonstrated by De Felice et al. The response to induction chemotherapy can be used as a biomarker of chemoresistance and can guide the intensification or de-escalation of treatment. Analyzing data obtained retrospectively from 445 patients with locally advanced HNC, including cancers of the oral cavity, oropharynx, hypopharynx and larynx, of which 52% received definitive chemoradiotherapy and 42% surgery followed by adjuvant treatment, Lee et al. identify similar two-year survival rates in the two groups analyzed. In the group of patients who received non-surgical treatment, Cisplatin was the most widely used concomitant agent administered weekly. For where induction chemotherapy preceded concurrent radio-chemotherapy, docetaxel, cisplatin and fluorouracil were the most commonly used agents. After analyzing the results, the authors conclude that for locally advanced HNC, both definitive non-surgical multimodal treatment, including concurrent chemoradiotherapy associated or not with induction chemotherapy, and surgical treatment followed by adjuvant therapy, are feasible options [47,48,49,50].

The recommendation to omit chemotherapy in patients >70 years or younger, especially >60 years with comorbidities during a possible Covid-19 pandemic outbreak is a suitable choice. Older age, comorbidities and limited benefit of concomitant chemotherapy addition, associated with the additional risk of intensive treatment in the case of a SARS-CoV-2 infection, make elderly patients possible cases with a high risk of ICU admission, mechanical ventilation or even death. A cisplatin dose ≥200 mg/m^2^ is considered an independent prognostic factor for OS, with the strongest evidence coming from the treatment of nasopharyngeal cancers. Gundong et al. believe that weekly Cisplatin is equivalent to a three weekly Cisplatin scheme only if a cumulative dose of 200 mg/m^2^ is reached. In a phase III study Noronha et al. demonstrate the superiority of administration of 100 mg/m^2^ at three weeks in terms of local control but not in OS, the weekly dose of Cisplatin in this case being 30 mg/m^2^. Increased hospitalization rate (31.1% vs. 11.3%) but also higher rate of adverse events was observed when a three weekly scheme was preferred (84.6% vs. 71.6%) [48,49,50,51,52,53,54,55,56,57,58].

Given the indication not to discontinue treatment in the event of possible contamination with the new coronavirus, it is possible that any radiotherapy department will treat patients with moderate and mild symptoms during the pandemic outbreak. In this respect hygiene rules and measures of social distancing by limiting the time spent by patients in the department, triage and the use of alcohol-based solutions for hand disinfection and chlorine-based solutions for surface disinfection are measures considered standard in most departments. Dividing staff into teams that communicate with each other without direct contact, using audio-video communication systems between team members, and limiting to the maximum the number of healthcare workers who will contact patients, are also regulations intended to reduce risk of infection. Alterio et al. describe the experience of a radiotherapy department regarding the treatment of HNC in Milan, Italy, a region that was in February 2020 the first European epicenter of the Covid-19 pandemic. Special regulations have been proposed for the category of patient considered at increased risk of transmitting and contacting SARS-CoV-2. In addition to general preventive measures, all CT-simulations were scheduled on a dedicated day, all treatment sessions of HNC patients were performed in the morning, and thermoplastic masks and mouthpieces were disinfected with alcohol-based solutions. The radiotherapy team from Wuhan, proposed the use of this mask during treatment sessions, but in order not to create breathing difficulties for the patients a window was cut into the thermoplastic mask [59,60,61].

For early laryngeal cancer T1N0, a hypo-fractionated regimen of 50 Gy/16 fractions and a 55 Gy/20 fractions regimen for T2N0 disease are the preferred option of ESTRO-ASTRO experts. Even if the evidence of hypo-fractionation is limited in locally advanced cancers concerning SIB technique (using the same total dose in a smaller number of fractions), experts propose, in a scenario of limiting resources, several hypofractionation schemes: 55 Gy/20 fractions, 62.5–64 Gy/25 fractions and 54 Gy/18 fractions [22,61,62,63].

Even if Jacinto et al. demonstrate a similar rate of toxicity in concurrent chemoradiation protocol with 35 mg/m^2^ weekly Cisplatin and doses of 55 Gy/20 fractions to the gross tumor and 44–48 Gy/20 fractions for subclinical disease, concurrent administration of chemo-radiotherapy is preferred if a moderate hypo-fractionation is chosen. HPV- oropharyngeal cancers show significant differences in evolution and prognosis, being diagnosed at a younger age compared to HPV+ oropharyngeal cancers. Patel et al. proposed treatment de-escalation strategies for HPV+ oropharyngeal cancers, demonstrating non-inferiority of a low-dose treatment (60 Gy) and weekly Cisplatin, but in the study group only 16% of patients had >N2b disease. Smoker status (shown to have prognostic and predictive value in HPV-oropharyngeal cancers) has not been considered in this study. One of the characteristics of this disease is a favorable response to initial treatment but the rate of distant metastases is similar to that of HPV- oropharyngeal cancers. The inferior results given by the replacement of Cisplatin with Cetuximab in this category of patients and the increased risk of distant metastases highlight the role of systemic treatment in this subcategory of H&N [64,65,66,67,68].

Sharing the experience of The Princes Margaret Cancer Center on the use of moderate hypofractionation (60 Gy in 25 fractions over five weeks), the authors of a retrospective study that included 61 and 263 cases of head and neck squamous cell carcinoma HPV+ and HPV− concluded that During the Covid-19 pandemic, moderate hypofractionation may replace standard treatment with definitive chemotherapy in the cases of HPV+ T1-T3N0-N2c and HPV− T1-T2N0 and selected stage III patients (according to TNM-7) [39].

## 5. Conclusions

Locally advanced HNC cancer is a unique challenge for cancer management in the Covid-19 scenario. The negative consequences of delaying radio-chemotherapy make it necessary to highly prioritize these patients, indicating the continuation of therapy even if SARS-CoV-2 infection is confirmed and patients do not have severe symptoms and additional risk factors. Induction chemotherapy is not considered the optimal option in this situation because of the risk of immunosuppression even though, in selected groups, the taxane, platinum fluorouracil TPF regimen may bring benefits. For an early scenario the standard chemo-radiotherapy is the preferred option, the SIB technique can reduce the overall treatment time. For a late scenario with a possible limited technical and human resources, hypo-fractionation with possible omission of chemotherapy in certain situations is recommended. Although MACH-NC did not demonstrate the superiority of induction chemotherapy over concurrent chemo-radiotherapy, the TPF regimen could be considered even in the case of a possible Covid-19 pandemic scenario, but Cisplatin substitution with Carboplatin is risky due to the hematological toxicity associated with immunosuppression. Any therapeutic decision must be based on a careful analysis in MDT to assess the risks and the need for surgical procedures in the context of severely limited resources. Moderate hypofractionation alone with a total dose of 60 Gy in five weeks, with a daily dose of 2.4 Gy per fraction can be considered in cases with a low risk of distant failure. In cases where concurrent chemoradiotherapy is chosen, a SIB-IMRT or SIB-VMAT regimen with 70 Gy in 33 fractions may be considered.

## Data Availability

Data analyzed in this study were a re-analysis of existing data, which are openly available at locations cited in the reference section.
